# 肺移植术后肺癌1例报告并文献复习

**DOI:** 10.3779/j.issn.1009-3419.2011.01.16

**Published:** 2011-01-20

**Authors:** 烨铭 王, 静瑜 陈

**Affiliations:** 214023 无锡，南京医科大学附属无锡市人民医院胸外科和肺移植科 Department of General Toracic Surgery, Department of Toracic Transplant, Wuxi People's Hospital Afliated to Nanjing Medical University, Wuxi 214023, China

**Keywords:** 肺移植, 肺肿瘤, 肺间质纤维化, 免疫抑制, Lung transplantation, Lung neoplasms, Idiopathic pulmonary fbrosis, Immunosuppression

## Abstract

**背景与目的:**

肺移植是治疗终末期肺部疾病的有效手段，然而对肺移植术后肺癌却缺乏了解。我们通过对1例肺移植术后肺癌患者临床资料的报道，并结合相关文献复习，以提高对肺移植术后肺癌的认识、诊断及治疗水平。

**方法:**

2007年5月我院为一例65岁、术前诊断为两肺特发性肺间质纤维化（idiopathic pulmonary fibrosis, IPF）的男性患者在体外膜肺氧合（extracorporeal membrane oxygenation, ECMO）辅助下成功进行了右侧单肺移植，患者术后46 d恢复良好出院。术后免疫抑制方案为他克莫司（Tac）+吗替麦考酚酯（骁悉）+类固醇激素。出院后患者定期随访。

**结果:**

在移植术后13个月随访确诊为非移植侧左肺小细胞肺癌伴多发骨转移。患者给予依托泊苷联合顺铂（EP方案）化疗4次症状有所缓解，患者在诊断肺癌后11个月死亡。

**结论:**

肺移植术后肺癌严重影响移植患者远期存活，慢性阻塞性肺病（chronic obstructive pulmonary disease, COPD）、IPF、吸烟史及免疫抑制剂等为其危险因素，为改善预后，需要早期诊断及早期治疗。

## Abstract (en)

**Background and objective:**

Lung transplantation is an efcient therapeutic option for patients with end-stage pulmonary diseases, but less is known about lung cancer afer lung transplantation. The aim of this study is to improve the awareness, diagnosis and treatment of bronchogenic carcinoma after lung transplantation with a case report and related literatures.

**Methods:**

We reported a 65-year-old male with idiopathic pulmonary fbrosis (IPF) who underwent right lung transplantation under extracorporeal membrane oxygenation (ECMO) support in May 2007 in our hospital. The patient recovered smoothly and discharged from the hospital 46 days afer the procedure with regular follow-up. Immunosuppression therapy was triple drug maintenance regimen including tacrolimus (Tac), mycophenolate mofetil (MMF) and steroids.

**Results:**

Small cell lung cancer in the lef lung with multiple osseous metastases was found 13 months afer the lung transplantation. Symptoms were relieved a bit by administering chemotherapeutics (etoposide and cisplatin) for 4 cycles. However, the patient was succumbed to his illness within 11 months afer the diagnosis of lung cancer.

**Conclusion:**

Lung cancer afer lung transplantation has been suggested as one of causes of late mortality with the risk factors such as chronic obstructive pulmonary disease (COPD), IPF, cigarete smoking history and immunosuppression *etc*. Early diagnosis and treatment are very important to improve the prognosis.

肺移植术后肺癌严重影响移植患者的远期存活^[1]^。现将我院1例肺移植术后肺癌的病例报道如下，并行文献复习，以提高对肺移植后肺癌的认识、诊断及治疗水平。

## 1 临床资料

受者是一例65岁男性，有“特发性肺间质纤维化（idiopathic pulmonary fibrosis, IPF）”病史7年，吸烟史40余年，平均1包/天，粉尘接触史2年，入院时需吸氧6 L/min。在院时高分辨率CT（high resolution computerized tomography, HRCT）检查示：两肺IPF，呈蜂窝样改变（图 1A）。术前诊断为IPF继发性肺动脉高压、右心功能不全。移植术前供、受者ABO血型相符，供肺的维持、获取及保存见文献^[1]^，患者在麻醉、气管插管后，经右侧股动、静脉插管，在体外膜肺氧合（extracorporeal membrane oxygenation, ECMO）转流辅助下经右侧前胸切口完成右侧的单肺移植^[2]^。手术顺利完成，手术结束即撤除ECMO辅助。术后予“他克莫司+吗替麦考酚酯（骁悉）+类固醇激素”的免疫抑制方案预防排斥反应。患者术后46 d恢复良好出院，出院前复查CT见图 1B。患者定期门诊复查，术后2个月发生一次急性排斥反应，予积极治疗好转。在移植手术后13个月因咳嗽痰血行胸部CT发现左下肺团块影（图 1C），行支气管镜检查发现左下支气管口新生物，活检病理结果为小细胞肺癌。骨扫描及MRI检查发现已有多发胸、腰椎体转移（图 1D）。患者予以EP方案化疗4周期症状有所好转，但最后在确诊肺癌11个月后死亡。

**1:**
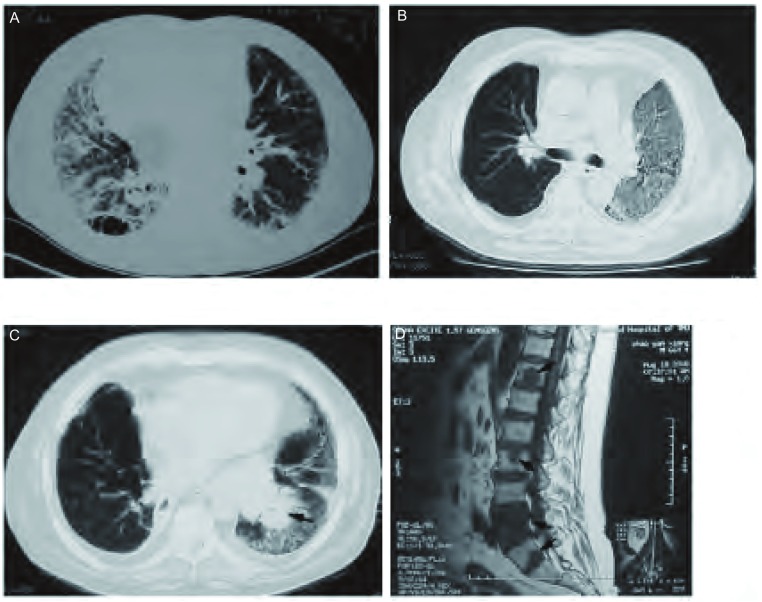
患者临床影像学特征。A：术前CT扫描显示两肺弥漫性纤维化，呈蜂窝样改变，尤其以右肺明显；B：术后一月复查CT显示移植肺扩张良好，左肺弥漫性纤维化加重；C：肺移植术后13个月复查CT显示左肺弥漫性纤维化，左下肺心缘团块影；D：同期行MRI，显示有多发骨转移（T12、L3、L5、S1）。 Clinical radiologic features of the patient. A: Preoperative HRCT showing predominant reticular abnormality with honeycombing, particularly in the right lung; B: Postoperative HRCT (1 month) showing satisfactory reexpansion of the transplanted lung and progression of lung fibrosis in the left native lung; C: Postoperative HRCT (13 months) showing fibrosis in the left lung and a pulmonary mass in the left lower lobe (cardiac border, arrow); D: Postoperative MRI (13 months) showing multiple osseous metastases in the vertebral column (T12、L3、L5、S1, arrows).

## 2 讨论

### 2.1 肺移植后肺癌的发病率

早在20世纪，Penn^[3]^收集了移植后并发肿瘤患者的资料，其数据显示，除了慢性排斥反应，影响实体器官移植（包括肺移植）患者长期存活的最主要因素为移植后新生恶性肿瘤。自1983年多伦多肺移植组成功完成了人类首例长期存活的肺移植以来^[4]^，肺移植已在全球范围展开。至2008年6月底^[5]^全世界共完成单、双肺移植29, 732例，且近几年以超过2, 300例的数目增长，据国际心肺移植协会（International Society for Heart and Lung Transplantation, ISHLT）的统计，肺移植术后新生恶性肿瘤导致的死亡虽然不是主要原因，但仍占据一定的比例，其发生率在术后30天、1年内、1年-3年、3年-5年、5年-10年、10年以上分别为0.3%、5.3%、8.4%、10.1%、12.2%和13.5%。

McAdams等^[6]^报道肺移植后肺癌发生率为3/111（2.7%），IPF和慢性阻塞性肺病（chronic obstructive pulmonary disease, COPD）患者行肺移植后肺癌发生率分别为1/15（6.67%）、2/86（2.33%）。Collins等^[7]^则报道上述3项数值分别为24/2168（1.11%）、6/147（4.08%）、18/859（2.10%）。另有报道^[8]^称COPD患者在单肺移植后发生肺癌的概率为5.15%，自确诊到死亡的时间为10.8周（1周-60周），1年内致死率为75%。

我院移植中心2002年9月-2009年12月共完成移植86例，其中IPF患者38例。目前仅发现1例IPF患者患肺移植术后肺癌，总发病率为1/86（1.16%），IPF患者中发病率为1/38（2.63%），与国外文献报道的发病率相似。然而，由于受到肺移植数的限制，因此对肺移植后肺癌发生率的估计还需要肺移植数的增加以及长期的随访研究。

### 2.2 肺移植后肺癌的原因分析

有学者^[9]^认为，肺移植术后发生肺癌的因素还和受体自体肺的病种以及单、双肺移植术式有关，COPD和间质性肺病（石棉肺、矽肺、IPF等）均是肺癌的危险因素。就目前所知，COPD的病因如长期吸烟、理化因素刺激及感染等，可能在癌症形成过程中起到协同作用；IPF导致的免疫炎症损伤及本身在形成和发展过程中异常活化癌基因，亦有可能引起肺癌。国外有综述^[10]^显示IPF患者的肺癌发病率约为17.3%。然而，COPD或IPF的发病是否同时对癌症的产生起到刺激作用，目前尚不能确定，还有待进一步研究。Dickson等^[11]^研究发现，6.9%的单肺移植患者在移植后出现原发性肺癌，而双肺移植患者的发生率为0。并且认为，肺移植后发生原发性肺癌的危险因素包括：年龄的增长、吸烟史 > 60包年以及单肺移植（与双肺移植对比）。Mathew等^[12]^的观点与之相似。

本例患者有长达40余年的吸烟史，平均1包/天，虽与Dickson等^[11]^所言60包年的吸烟史有出入，然而，40包年也是可观的，而吸烟本身也是肺癌的一大诱发因素。此外，该患者另有2年的粉尘接触史，因此，不能完全排除其是否与肺癌的发生有关联。同时，患者行单肺移植，有自体肺的存在，这与国外报道^[11, 12]^一致。

免疫抑制剂在心肺移植中的成功应用为终末期肺血管疾病及其它某些难治性心肺疾病患者带来了曙光。然而，识别和及时清除衰老恶变的自体组织细胞是人类免疫系统的一大功能，移植患者在术后长期使用免疫抑制剂，使机体的免疫系统受损，造成免疫抑制状态，免疫系统对突变细胞及病毒的免疫监视作用减弱或消失，因此此类患者易致肿瘤基因激活或遭受致瘤病毒感染。而相比肾移植，心脏或肺移植的患者，术后需要更大剂量的免疫抑制剂，故发生肿瘤的危险性更高^[13]^。另外某些免疫抑制剂的直接致癌作用，可能会进一步导致肿瘤发病率的增加。而有学者^[14]^则认为免疫抑制剂并非致癌因素，他们的研究显示，年龄较小的受者即使有长期（> 10年）免疫抑制剂使用史，也并未新生肺癌；其次，也并未发现无吸烟史者在术后新生肺癌。然而，他们的样本含量偏小，因此对于免疫抑制剂的应用及肺癌的联系有待更深的研究。

近年来又有研究发现，并非所有的免疫抑制剂都会增加肿瘤的发生率，相反有些甚至具有抗肿瘤的性质，例如雷帕霉素（西罗莫司，SRL）^[14]^、FTY720^[15]^、咪唑立宾^[16]^、吗替麦考酚酯（MMF）^[17, 18]^及来氟米特^[18]^均有明显的抗肿瘤效应。采用这类免疫抑制剂，可取得抗排异和抗肿瘤的双重作用，对预防肺移植术后肺癌的意义有待进一步研究。

### 2.3 早期诊断及治疗

在影像学上，肺癌可表现为边界清楚的孤立小结节、多发性结节、肿块或阻塞性肺炎^[6, 19]^，且对于单肺移植者，均在自体肺发现病灶。普通X线胸片和CT均能发现肺部的病变，采用何种检查方法需要权衡肺癌发生的可能性。另外，考虑到影像检查带来的放射线危害，间隔多长时间行一次X线或CT还有待进一步研究。也有学者^[20]^对CEA与肺移植后患恶性肿瘤的相关性进行了研究，他们发现，术后发生癌症的患者移植前CEA平均水平较正常人高，但与未发生癌症的肺移植患者无明显差异。他们认为，CEA水平或许只是基础疾病的标记，并不能预测移植后存活或恶性肿瘤的发生。

鉴于移植患者免疫功能低下，有更高的恶性肿瘤易患性，因此需要临床医生和患者及其家属对此加以重视。无论是临床复诊还是日常生活，都不应放过任何异常，以便尽可能早期诊断。

目前认为，发现肿瘤后首先应考虑以根治性手术切除病灶为主的综合治疗。肺移植后肺癌，其治疗原则与一般肺癌治疗相似^[21]^，同时还应考虑到肺移植术后患者的特殊性，有必要调整免疫抑制剂的治疗方案，并兼顾移植物功能的维持。我们认为，首先可将Tac更换为SRL，其次，在使用最小剂量维持移植物功能正常的前提下，尽早手术；若失去手术机会，则应进行放、化疗，尽量提高患者生存质量。移植肺的存在并非是肺癌手术的绝对禁忌症，只是有必要对全身情况进行更全面的评估。本例患者最早是在CT上发现自体肺的占位，然而，发现时肿瘤已有转移，失去了手术机会，只能予以化疗，而不能切除病灶，故远期生存受到限制。

总之，肺移植已日益成为治疗终末期肺病的唯一方法，提高对肺移植后肺癌的认识、诊断及治疗水平，有利于提高肺移植患者的远期生存率。
